# Insulator Defect Detection in Complex Environments Based on Improved YOLOv8

**DOI:** 10.3390/e27060633

**Published:** 2025-06-13

**Authors:** Yuxin Qin, Ying Zeng, Xin Wang

**Affiliations:** 1 School of Electrical and Information Engineering, Hunan University of Technology, Zhuzhou 412007, China; y.qin.1@research.gla.ac.uk; 2 School of Computer Science, University of Glasgow, Glasgow G12 8QQ, UK; 3 School of Computer Science, Hunan University of Technology, Zhuzhou 412007, China; m21085400026@stu.hut.edu.cn

**Keywords:** insulator defect detection, improved YOLOv8, C2f_DSC network, feature fusion, entropy

## Abstract

Insulator defect detection is important in ensuring power systems’ safety and stable operation. To solve the problems of its low accuracy, high delay, and large model size in complex environments, following the principle of progressive extraction from high-entropy details to low-entropy semantics, an improved YOLOv8 target detection network for insulator defects based on bidirectional weighted feature fusion was proposed. A C2f_DSC feature extraction module was designed to identify more insulator tube features, an EMA (encoder–modulator–attention) mechanism and a BiFPN (bidirectional weighted feature pyramid network) fusion layer in the backbone network were introduced to extract different features in complex environments, and EIOU (efficient intersection over union) as the model’s loss function was used to accelerate model convergence. The CPLID (China Power Line Insulator Dataset) was tested to verify the effectiveness of the proposed algorithm. The results show its model size is only 6.40 M, and the mean accuracy on the CPLID dataset reaches 98.6%, 0.8% higher than that of the YOLOv8n. Compared with other lightweight models, such as YOLOv8s, YOLOv6, YOLOv5s, and YOLOv3Tiny, not only is the model size reduced, but also the accuracy is effectively improved with the proposed algorithm, demonstrating excellent practicality and feasibility for edge devices.

## 1. Introduction

Insulators are used to fix conductors to ensure smooth electricity transmission. However, they are easily damaged due to exposure to adverse outdoor weather conditions [1]. Traditional research usually involves staff operating drones to capture images and videos of insulators, and then manually analyzing them to identify defects and potential hazards [2]. AI-driven and information theory-based solutions have become indispensable in modern industries over the past decade [3,4,5]. Automatic defect detection as an efficient method has been applied to many areas, which not only improves production efficiency but also protects employees from potential hazards [6,7,8]. Currently, many methods related to deep-learning technology for insulator defect detection have been proposed [9]: one-step algorithms with regression, such as SSD (single-shot multibox detector) [10,11], YOLO series [12,13,14,15,16], etc.; two-step algorithms with candidate regions, such as R-CNN [17], Fast R-CNN [18,19], Mask R-CNN [20]; and hybrid methods, such as YOLO-HMC [6], YOLOu-Quasi-ProtoPNet [21], etc. The YOLO series algorithms have become some of the main algorithms in object detection applications in recent years. Wu et al. [22] proposed an improved YOLOv3 algorithm for insulator defect detection that accelerates the calculation speed by replacing the Darknet-53 structure in the backbone feature extraction network with the MobileNetV1 structure. However, its accuracy is relatively low. Li et al. [23] added a SimAM attention mechanism based on YOLOv3, effectively improving the feature extraction ability of images without changing the original feature pyramid network. However, this method cannot accurately recognize targets in complex environments. To better capture effective information, Xiao et al. [24] integrated a GAM attention mechanism and an ASFF adaptive feature fusion mechanism in the YOLOv5, but the accuracy of its object detection in complex environments is relatively low. Wang et al. [25] proposed an insulator defect detection method based on AMC-YOLOX-s, inserting a CA coordinate attention mechanism into CSPDarkNet to improve the classification and localization ability of insulators. Zou et al. [26] integrated a CA attention mechanism and BiFPN into the YOLOv7 algorithm and used data augmentation to improve the model performance in detecting insulator defects in complex environments, but the detection speed was slow. Jia et al. [27] integrated depth-wise separable convolution, point-wise convolution, and an ECA attention mechanism into YOLOv5 to improve the shallow network’s ability, greatly reducing the model’s complexity and solving its embedding problems in mobile devices. However, the model’s accuracy is still low.

Although the insulators can be detected well using the above algorithms, it is difficult to recognize insulator defect targets in complex environments in real time. This needs to find the trade-offs among model size, model detection speed, and accuracy, and execute embedded applications on edge devices [28]. To deal with the above problems, an improved YOLOv8 target detection network for insulator defects based on bidirectional weighted feature fusion is proposed. The main contributions of this paper are as follows:

(1) To tackle the intricate morphology of insulators, a new C2f_DSC network module was designed, combining a dynamic snake convolution (DSConv) kernel [29] with entropy-regulated feature compression. This convolution kernel structure can better capture the basic features of insulator defect areas, improve the perception ability of subtle defect targets, and enhance the robustness and accuracy of the algorithm.
(2) To address the entropy imbalance and feature fusion in insulator defect detection, BiFPN [30] was improved by adjusting its parameters and connections for multi-scale feature fusion, prioritizing defect-related features, and thereby enhancing recognition accuracy.
(3) The EMA mechanism [31] was integrated into the model, incorporating high-information–content features and performing weighted average processing on feature maps during training, highlighting key information, and improving the model’s attention to insulator defect areas. This integration enhances the model’s self-adaptive feature adjustment ability, enabling it to maintain good performance under different conditions.
(4) The EIOU loss function [32] was applied to YOLOv8. Compared with conventional CIOU (Complete Intersection over Union) [33], it explicitly incorporated geometric discrepancies between the target and the anchor boxes and further minimized geometric differences (e.g., center distance and aspect ratio) in a statistically guided manner, thereby improving the model’s convergence speed, accuracy, and stability.

These improvements form a unified entropy-driven framework, achieving synergistic optimization and enhancing feature extraction capability, accurate recognition, and localization ability for insulator defect targets in complex environments.

The remainder of this article is organized as follows: An improved YOLOv8 is proposed in Section 2. The performance of the improved YOLOv8 and its competitors is compared in Section 3. Finally, conclusions are drawn in Section 4.

## 2. Materials and Methods

### 2.1. YOLOv8 Algorithm

The YOLOv8 algorithm was released by Ultralytics (Frederick, MD, USA) in 2023 [15]. Its structure includes a backbone network, an anchor-free network, and a new loss function, and it achieves entropy-regulated feature optimization through three key mechanisms: information compression in the backbone network, entropy balancing within the feature pyramid, and entropy minimization at the detection head. Therefore, YOLOv8 is extremely efficient and can run on various platforms, from CPU to GPU. It is easy for users to switch between different YOLO versions.

YOLOv8′s backbone network is similar to YOLOv5. It is based on the idea of the CSP network structure and the ELAN structure in YOLOv7 [14]. C2f module is formed by combining C3 and ELAN (efficient lightweight attention network), which enables YOLOv8 to better capture rich features while maintaining its lightweight design. The backbone network of YOLOv8 still uses the most popular SPPF (spatial pyramid pooling fast) module, sequentially passing three Maxpools with a size of 5 × 5, and then connecting them between layers. This ensures the objects’ accuracy at different scales while ensuring their lightweight design.

**Figure 1:**
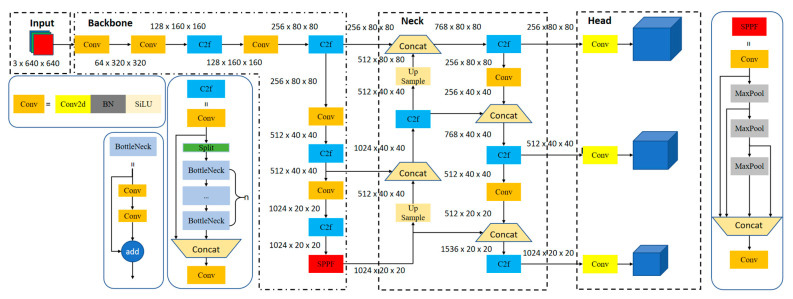
YOLOv8 network structure.

In the neck region, PAN [34] + FPN [35] are still the main feature fusion methods used by YOLOv8, which includes two upsampling, multiple C2f modules, and decoupling head structures. This architecture achieves multi-scale entropy balancing through complementary integration of high-level (low-entropy) and low-level (high-entropy) features while optimizing entropy-regulated information flow across network hierarchies. In YOLOx [36], the decoupling head was applied to the neck by YOLOv8. YOLOv8 currently has five versions. These versions differ in terms of the depth and width of the networks. The YOLOv8n network structure is shown in Figure 1. It includes four core components: input, backbone feature extraction network, feature fusion network (neck), and prediction (head). The input mainly includes image data augmentation, image resizing, and adaptive anchors. The neck mainly includes the backbone network, feature pyramid, and loss function. The head mainly includes prediction box generation, prediction box filtering, and output.

### 2.2. Improved YOLOv8 Target Detection Network for Insulator Defects

**Figure 2:**
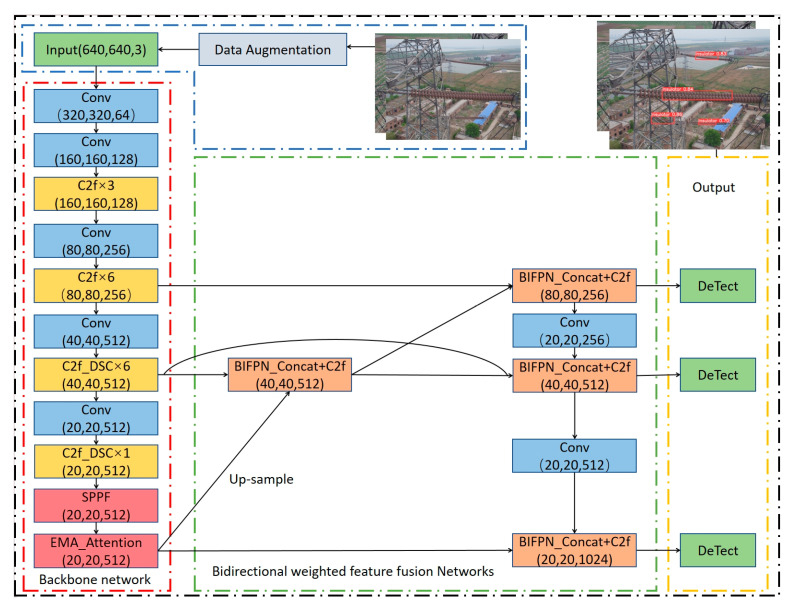
Improved YOLOv8 network structure for insulator defect detection.

An improved YOLOv8 target detection network for insulator defects based on bidirectional weighted feature fusion is proposed, as shown in Figure 2. Data augmentation was first implemented to enhance dataset diversity. A C2f_DSC module was specifically designed on the original backbone network to better adapt to complex insulator morphological variations. Concurrently, a bidirectional weighted feature fusion mechanism and a novel composite loss function were integrated to optimize target detection efficiency while balancing computational demands and memory constraints. These architectural enhancements enable precise capture of subtle defect signatures, thereby achieving accurate identification of both insulators and defect targets in challenging real-world scenarios.

#### 2.2.1. Entropy-Driven Information-Guided Augmentation

To address the limited defect samples in the CPLID dataset, we developed an information–theoretic augmentation framework that generates semantically meaningful synthetic samples. The core principle involves selecting augmentation strategies (e.g., CutMix, Mosaic) to maximize mutual information (MI) between input images and defect classes:

(1) I(X;Y)=H(X)−H(X∣Y)

where *XX* is the input image and *YY* is the defect category.

#### 2.2.2. C2f_DSC Module

**Figure 3:**
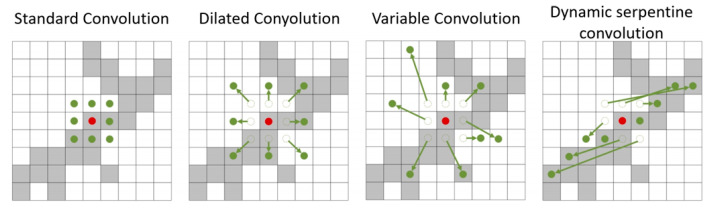
Receptive fields of different convolutions (Green dots: sampling point positions; red dot: center position).

To extract local weak structural features, as well as diverse global morphological features at various scales, DSConv [29] was introduced and stacked multiple times to expand the receptive field ranges. DSConv is a convolutional neural network (CNN) technique in deep learning aimed at addressing certain limitations in convolution operations, leveraging entropy-driven adaptive mechanisms to enhance the network’s capacity for irregular data. Specifically, it dynamically adjusts the shape and size of convolutional kernels based on local feature entropy and data irregularity, optimizing kernel configurations through information–theoretic prioritization of high-information regions. The principle of increasing the receptive field through DSConv convolution is as follows:

In the standard 2D convolution coordinate system *K* with center coordinate *K_i_* = (*x_i_*, *y_i_*), a conventional 3 × 3 convolution kernel with dilation rate of 1 is defined as *K* = {(*x* − 1, *y* − 1), (*x* − 1, *y*),…,(*x* + 1, *y* + 1)}. Inspired by deformable convolution [37], we introduce an entropy-optimized deformation offset *∆* to the dynamic snake convolution, where the elastic deformation of kernels along x–y axes is guided by minimizing the feature spatial entropy *H*(*p*). Specifically, the selection process for each grid position *K_i_*_±*c*_ = (*x_i±c_*, *y_i_*_±*c*_) (where *c* = {0, 1, 2, 3, 4} is the horizontal distance from the center grid) incorporates the principle of maximizing local mutual information *I*(*X*;*Y*), ensuring the dynamic adjustment of offset *∆* = {*δ* | *δ* ∈ [−1, 1]}, and following the information bottleneck theory. This entropy-constrained deformation mechanism guarantees that the accumulation process *K_i_*_+1_ = *K_i_* + *∆* consistently focuses on low-entropy, high-information-density regions. Not only does this approach enable each pixel in deeper network layers to cover wider input areas for capturing global features, but it also significantly improves information extraction efficiency by suppressing computational redundancy in high-entropy noise regions. As shown in Figure 3, compared to traditional convolutions, the resulting receptive field demonstrates distinct entropy-adaptive characteristics, exhibiting superior information-capturing capability in complex scenarios.

**Figure 4:**
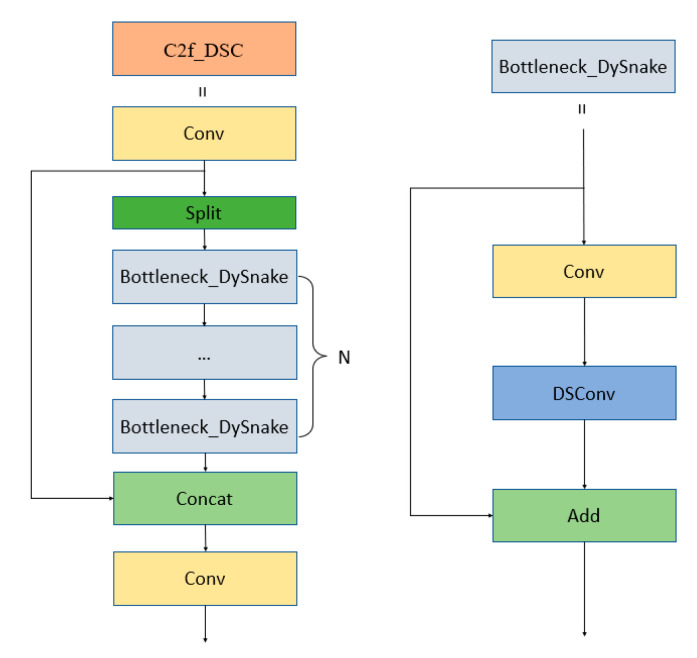
Designed C2f_DSC module structure.

Compared with other convolution methods, dynamic snake convolution performs better in extracting features of tubular structures. Its design concept helps to more accurately and comprehensively capture feature information with tubular structures. The final designed C2f_DSC module structure is shown in Figure 4. A 1 × 1 convolution was employed to alter the number of channels in the input features, and then the split operation was used to replace the 1 × 1 convolution for feature segmentation. More skip connections were also used to decrease the parameters while extracting richer multi-scale insulation sub-features, enabling recognition of the complex structural and morphological features of insulators.

#### 2.2.3. EMA Mechanism

**Figure 5:**
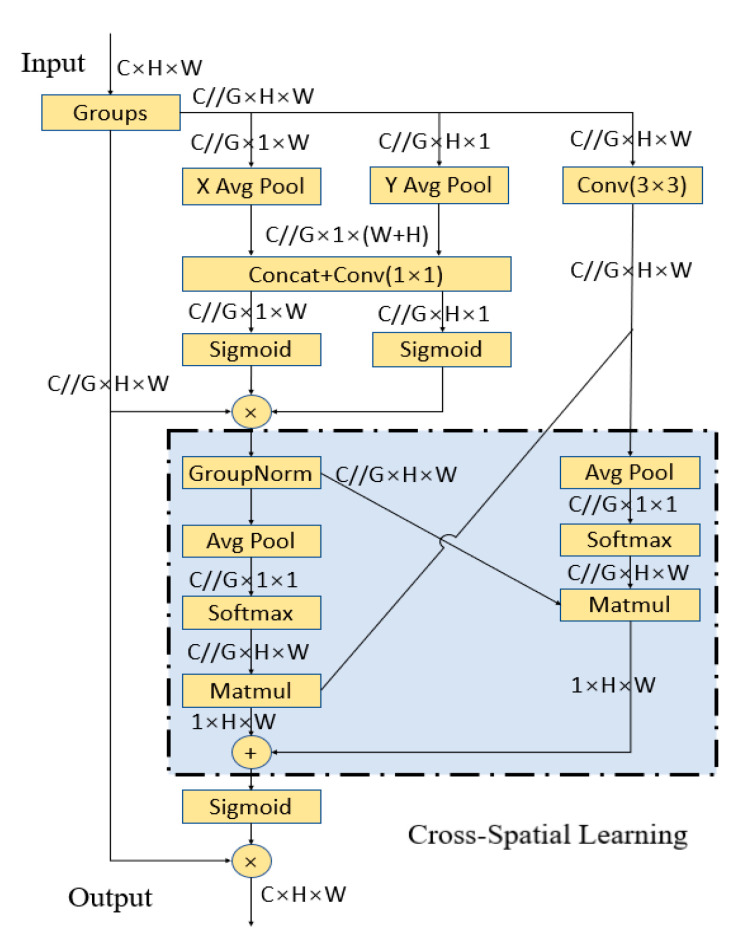
EMA mechanism module structure.

The attention mechanism plays an important role in computer vision, as it helps models focus on the most critical local information in the images, especially for detecting target-related features. Therefore, high-information features can be selected through mutual information to optimize the network architecture or design attention mechanisms. Here, an EMA mechanism was incorporated into the final stage of the backbone network, helping the model concentrate on salient visual features, thereby improving the model’s performance. Moreover, the local cross-channel interactions in each parallel sub-network were established in the EMA mechanism without channel dimensionality reduction, which helps the network avoid excessive sequential processing and depth increases. The EMA mechanism module structure [31] is shown in Figure 5. “*G*” is the divided group. “*X Avg Pool*” is a horizontal global pool with one dimension. “*Y Avg Pool*” is a vertical global pool with one dimension. “*Avg Pool*” is the vertical global pool with two dimensions. *C* is the input channel number. *H* and *W* are the spatial dimensions of the input features. To obtain multi-scale spatial structural information together and respond quickly, 3 × 3 and 1 × 1 branches placed in parallel were adopted by EMA. Simultaneously, short-term and long-term dependencies were established effectively by the grouped features and multi-scale structure. The process of the EMA mechanism is as follows:

Firstly, the features are grouped. For any feature map, *X* ∈ *RC* × *H* × *W*, *X* = [*X*_0_, *X_i_*, … *X_G_*_−*1*_], where *X_i_* ∈ *RC*//*G* × *H* × *W*. Here, *X* is split into G sub-feature groups along the cross-channel dimension. This division helps the model learn different semantics. G≪C is taken, and the learned attention-weighted descriptors are used to focus on the regions of interest in each sub-feature, without sacrificing generality.

Secondly, parallel subnetworks are utilized. Multi-scale spatial information can be collected due to the neuron’s large local receptive field. Therefore, attention-weighted descriptors extracted through three parallel paths for grouped feature maps are used by EMA. One parallel route is 3 × 3 branches, and the other two routes are 1 × 1 branches, which are capable of capturing dependencies among all channels while also reducing computational complexity. Specifically, the channels are encoded along two spatial directions in the 1 × 1 branches through dual 1D global average pooling operations, while localized cross-channel dependencies are captured via 3 × 3 convolution in the 3 × 3 branches to increase the feature space.

Finally, cross-spatial learning is conducted. Two tensors are introduced by EMAs. One is the 1 × 1 branch output, and the other is the 3 × 3 branch output. The global spatial information in the 1 × 1 branch output is encoded using a two-dimensional global average pool. Before the joint activation mechanism of channel features, the smallest branch output is directly transformed into the corresponding dimensional shape to preserve the entire precise spatial position information. Within each group, the output feature maps are fused into dual spatial attention weights, and pixel-level pair-wise relationships are captured through the Sigmoid function, while the EMA module’s final output size remains the same.

#### 2.2.4. Improved Feature Fusion Layer

The traditional YOLO series employs PAN + FPN for feature fusion, integrating multi-scale features through horizontal connections and hierarchical pyramid structures. However, targets often exhibit challenges in complex scenes such as multi-scale variation, occlusion, and information entropy imbalance issues poorly addressed by conventional fusion methods. To enhance detection performance without inflating model size, we adopted a bidirectional weighted feature fusion network (BiFPN). BiFPN has the following advantages:

(1) Entropy-constrained adaptive weighting is introduced to optimize feature transmission. BiFPN maximizes mutual information flow across scales by dynamically learning feature weights based on local information density, improving feature expressiveness, and reducing information entropy loss during fusion.
(2) Fine-grained features are preserved by pruning low-information pathways (high entropy noise) while reinforcing high-information channels. This entropy-driven selection elevates detection accuracy.
(3) Feature weights are adjusted dynamically using entropy-minimized criteria, ensuring fusion prioritizes semantically rich (low entropy) regions. This flexibility adapts to target scale variations and occlusions in complex scenes.

**Figure 6:**
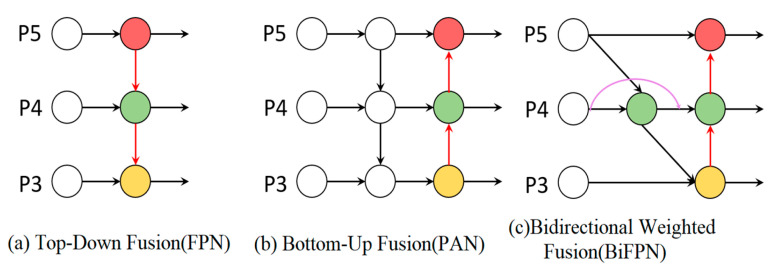
Different feature fusion structures. (**a**) FPN; (**b**) PAN; (**c**) BiFPN.

The model achieves higher detection accuracy and stability by integrating BiFPN, especially for targets with entropy-diverse scales (e.g., small objects in cluttered backgrounds). Different Feature fusion structures are shown in Figure 6.

#### 2.2.5. Improved Loss Function

To better evaluate the similarity between the target and the predicted box and more accurately measure the degree of matching between the predicted results and the actual targets, *EIOU* was adopted as the target loss function, which consists of overlap loss (*LIOU*), center distance loss (Ldis), and width-to-height loss (Lass). In addressing overlap loss and center distance loss, the similarity and the degree of matching were assessed by considering both their degree of overlap and the distance separating their center points. In addition, the width and height losses are directly minimized by *EIOU*. These optimization strategies can make model convergence more quickly and effectively improve model performance. The loss function equation for *CIOU* [33] is as follows:

(2) $$L_{CIOU}=1-IOU+\frac{\rho^{2}\left(b,b^{gt}\right)}{c^{2}}+\alpha \nu$$

(3) $$\alpha =\frac{\nu }{\left(1-IOU\right)+\nu }$$

(4) $$\nu ={\frac{4}{\pi^{2}}(arctan\frac{w^{gt}}{h^{gt}}-arctan\frac{w}{h})}^{2}$$

The loss function equation for *EIOU* [32] is as follows:

(5) $$L_{EIOU}=L_{IOU}+L_{dis}+L_{asp}\;=1-IOU+\frac{\rho^{2}\left(b,b^{gt}\right)}{c^{2}}+\frac{\rho^{2}\left(w,w^{gt}\right)}{c_{w}^{2}}+\frac{\rho^{2}\left(h,h^{gt}\right)}{c_{h}^{2}}$$

where *b* is the center point of the predicted box. $b^{gt}$ is the center point of the real box. $\rho^{2}\left(w,w^{gt}\right)$ is the width difference between the predicted box and the real box. $\rho^{2}\left(h,h^{gt}\right)$ is the height difference between the predicted box and the real box. $c_{w}$ and $c_{h}$ are the width and height of the smallest bounding box that covers the predicted box and the real box, respectively.

## 3. Results and Discussion

### 3.1. Experimental Dataset

**Figure 7:**
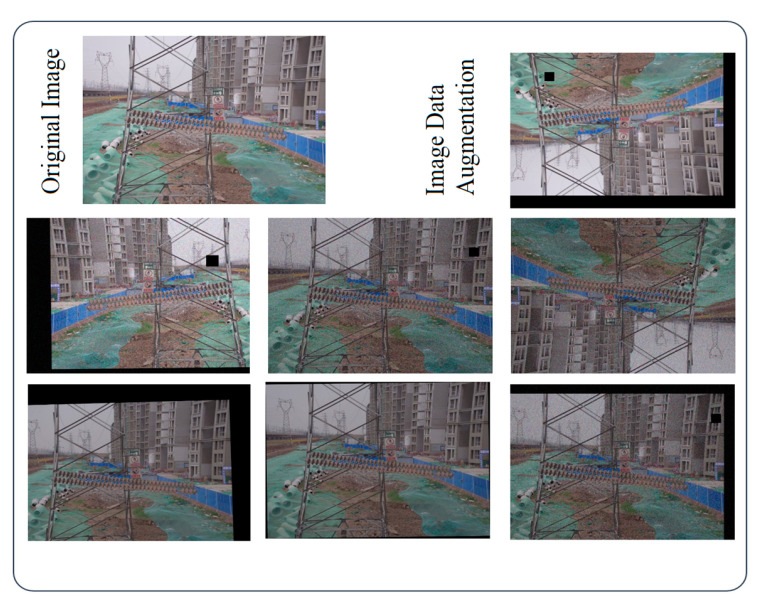
Insulator defect detection dataset augmentation.

To assess the effectiveness of the proposed model, we utilized a dataset from the China Power Line Insulator Dataset (CPLID) [38], which provides images of normal insulators and synthetic defective insulators captured by drones. The number of normal insulator images is 600, and the number of insulator images containing defects is 248. Due to the limited amount of data, we adopted data-augmentation methods [39,40] to increase the image samples. According to the idea of maximizing information gain, the data-augmentation methods included the following: (1) adding noise; (2) adjusting the brightness; (3) cutout; (4) rotation; (5) cutting; (6) translation; and (7) mirroring. These seven methods were randomly combined to expand the data to seven times its original size. Some sample cases are shown in Figure 7. Finally, the dataset was divided into a training set and a test set in a 9:1 ratio, with the training set consisting of 6106 augmented images and some original images, and the test set consisting of 678 original images.

### 3.2. Experimental Environment

The experimental environment is shown in Table 1, using PyTorch 1.8 as the deep-learning framework and a GPU for training. During the training process, all parameters were initially set to 200 iterations. The starting learning rate was 0.01, the batch size was 64, and the weight decay coefficient was 0.0005.

### 3.3. Evaluation Indicators

To evaluate the image detection model’s performance, multiple evaluation metrics were used for a comprehensive analysis. These indicators included mean accuracy (mAP), precision, recall, model parameter count (Params), weight file size (Size), and inference time (Inference Time). *Precision* was calculated as the proportion of predicted positive samples among all predicted samples, whereas *recall* was determined as the proportion of correctly predicted positive samples relative to the total actual positive samples, as illustrated in Equations (5) and (6), respectively.

(6) $$Precison=\frac{TP}{TP+FP}$$

(7) $$Recall=\frac{TP}{TP+FN}$$

where *TP* is the number of positive samples detected as true, *FP* is the number of negative samples detected as true, and *FN* is the number of positive samples undetected.

(8) $$AP=\int_{0}^{1}P(R)dR$$

(9) $$mAP=\frac{1}{m}\sum_{i=1}^{m}AP_{i}$$

where *AP* is the mean accuracy of each category, and *mAP* is the mean accuracy of all categories, as shown in Equation (7) and Equation (8), respectively. “mAP50” is the *mAP* when the intersection-over-union (IOU) threshold is 50%. “mAP50:90” is the mAP when the IOU threshold changes from 50% to 90%. The IOU threshold is typically used to measure the overlaps between the predicted bounding boxes and the actual bounding boxes. A threshold of 50% indicates that if the predicted bounding box overlaps with the actual bounding box by more than 50%, the predicted bounding box is considered correct [41].

### 3.4. Ablation Experiment

To verify the robustness of the proposed algorithm, ablation experiments were conducted on the CPLID dataset [38]. The essence of an Ablation Experiment is to quantitatively analyze the regulatory role of a specific component (e.g., module, layer, or connection) in an image information system by systematically removing or modifying it. The ablation experiment results are shown in Table 2, which demonstrates that the improved YOLOv8 algorithm outperformed the previous YOLO series algorithms. Although the weight size and time consumption increased slightly, the accuracy, robustness, and perceptual ability improved significantly, and, therefore, more accurate target localization and recognition were achieved at the expense of very few resources.

**Figure 8:**
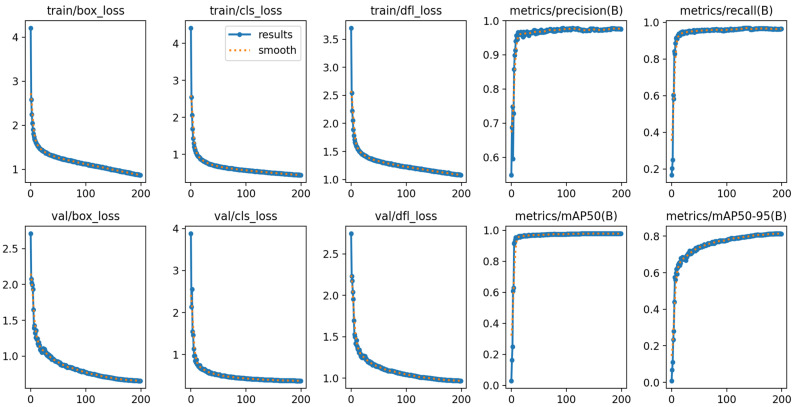
Training results of the improved YOLOv8 model for insulator defect detection (up/down pictures: evaluation indicators obtained with training/test set).

The training results of the improved YOLOv8 model are shown in Figure 8. The model was trained 200 times. As the training iterations increased, the loss functions of the training and testing sets rapidly decreased and eventually stabilized, while the precision, recall, mAP50, and mAP50:90 all rapidly increased and eventually stabilized. The mAP50 value remained stable at about 98.6%, demonstrating excellent performance during the training process.

### 3.5. Comparative Experiment

**Figure 9:**
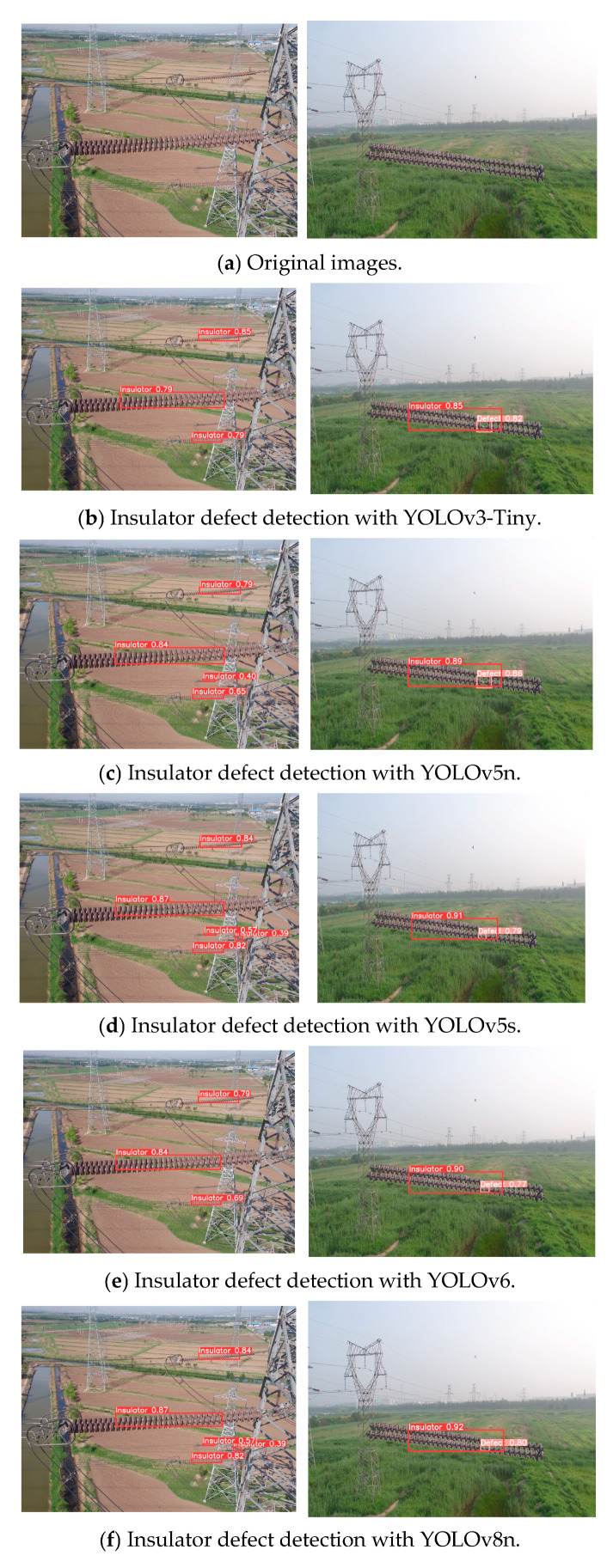

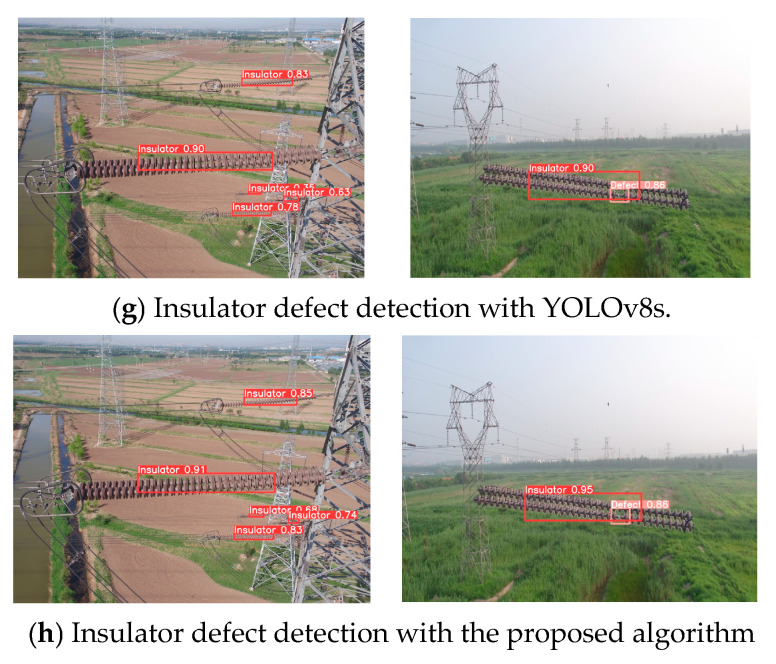
Comparative results with different YOLO algorithms for insulator defect detection.

To further validate the reliability of the proposed algorithm, multiple common YOLO series object detection models (YOLOv3 Tiny, YOLOv5n, YOLOv5s, YOLOv6, YOLOv8n, and YOLOv8s) were compared using the indicators mentioned above, and they were trained and tested on the same CPLID dataset. The comparison results are shown in Table 3. Higher accuracy was achieved while the parameter number and model size were reduced through the proposed algorithm, with a mAP value of 98.6%. Compared with YOLOv3 Tiny, YOLOv5n, YOLOv5s, YOLOv6, YOLOv8n, and YOLOv8s, the average detection accuracy was improved by 1.5%, 0.8%, 0.7%, 1%, 0.8%, 0.2%, and 0.8%, respectively, indicating that the improved model performs well in insulator defect target detection tasks. The comparative effects of the various models shown in Figure 9 verify that the enhanced YOLOv8 algorithm exhibits higher efficiency and accuracy and can correctly identify insulator targets even if they are in obscured states. Therefore, it is suitable for insulator defect detection applications in complex environmental scenarios.

**Figure 10:**
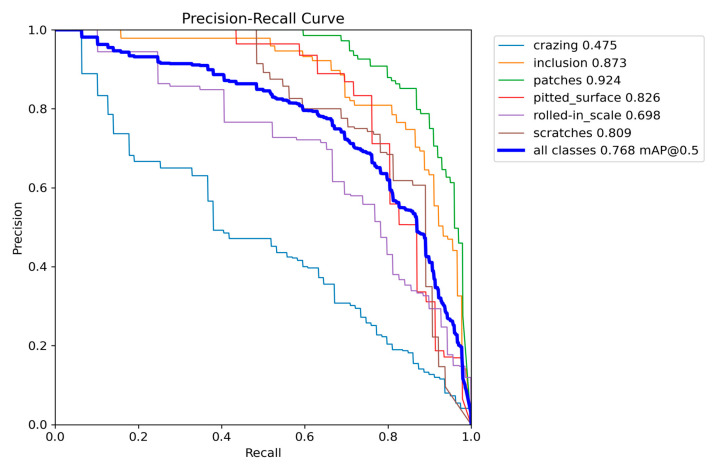
Precision/recall curves of improved Yolov8.

Experiments on steel surface defect detection were conducted to further demonstrate the practical applicability of the proposed model. The data is from the NEU-DET dataset (publicly released by Northeastern University in China), totaling 1800 images, and include six major categories of steel surface defects: “cracking”, “inclusion”, “patches”, “pitted_sturface”, “rolled-in-scale”, and “cracks”. Each category has 300 samples, including the training set of 270 samples and the testing set of 30 samples. The main results are shown in Table 4 and Figure 10, respectively. It can be seen that our model also has good adaptability, especially the snake convolution has better recognition of tubular objects, although it is designed for insulator defect detection. The improved C2f_DSC, BiFPN, and MEA enhance target detection ability. Therefore, our model will achieve good applications after in-depth development.

## 4. Conclusions

Aiming at the challenges of low accuracy, high latency, and large model size in insulator defect detection under complex environments, an improved YOLOv8 insulator defect detection algorithm based on bidirectional weighted feature fusion is proposed. A novel C2f_DSC structural module is designed to expand the receptive field for tubular insulators, and an EMA mechanism is incorporated at the end of the backbone network to extract multi-scale features from complex environments. Moreover, feature fusion is achieved using BiFPN, and the EIOU loss function is employed to accelerate model convergence. Experimental evaluations on the CPLID dataset demonstrate that the proposed model occupies only 6.40 M of storage space and achieves an average detection accuracy of 98.6%. Compared to YOLOv3 Tiny, YOLOv5n, YOLOv5s, YOLOv6, YOLOv8n, and YOLOv8s, the average detection accuracy of the proposed algorithm was improved by 1.5%, 0.8%, 0.7%, 1%, 0.8%, 0.2%, and 0.8%, respectively, while the model size and detection speed were maintained. To further demonstrate the practical applicability of the improved YOLOv8, experiments on steel surface defect detection were conducted. The results validate its excellent practicality and feasibility for edge devices, providing valuable experience for other object detection tasks. Future research will focus on the model’s lightweight design, further reducing its computational complexity and storage requirements, and balancing its real-time performance to adapt to a wider range of edge devices.

## Figures and Tables

**Table 1:** Experimental environment for insulator defect detection.

Experimental Environment	Environment Configuration
Frame	PyTorch 1.8
Language	Python 3.8
Operating system	Windows 10
Processor	Intel(R)Xeon(R)CPU E5-2680 v4, Intel, Santa Clara, CA, USA @2.4 GHz
GPU	24G NVIDIA Tesla M40, NVIDIA, Santa Clara, CA, USA

**Table 2:** Ablation experiment results with the CPLID dataset.

Methods	Data Augmentation	D2f_DySnake	EMA	BIFPN	EIOU	mAP50
1						94.7%
2	√					97.8%
3	√	√				98.0%
4	√	√	√			98.3%
5	√	√	√	√		98.5%
6	√	√	√	√	√	98.6%

**Table 3:** Comparative experimental results for insulator defect detection.

Name	Params	Size	Precision	Recall	mAP50	mAP50:95	ms
YOLOv3-Tiny	11.5 M	23.2 M	98.2%	94.9%	97.1%	82.0%	4.1 ms
YOLOv5n	2.39 M	5.02 M	97.5%	96.4%	97.8%	81.2%	3.9 ms
YOLOv5s	8.69 M	17.6 M	98.0%	96.3%	97.9%	82.4%	4.0 ms
YOLOv6	4.03 M	8.28 M	97.7%	96.6%	97.6%	81.0%	3.8 ms
YOLOv8n	2.87 M	5.97 M	98.3%	97.2%	97.8%	89.8%	4.0 ms
YOLOv8s	10.65 M	22.5 M	99.2%	97.5%	98.4%	90.6%	4.2 ms
Ours	3.05 M	6.40 M	99.2%	97.7%	98.6%	89.5%	4.1 ms

**Table 4:** Comparative experimental results for steel surface defect detection.

Name	Params	Size	Precision	Recall	mAP50	mAP50:95	FLOPS
YOLOv5s	2.5 M	17.6 M	62.0%	63.6%	68.3%	35.2%	7.2
YOLOv8n	3.01 M	5.97 M	71.1%	61.9%	68.3%	35.2%	8.2
Yolov8s	11.65 M	22.5 M	64.0%	65.4%	71.1%	38.3%	28
Ours	3.12 M	6.19 M	71.2%	69.9%	76.7%	42.0%	8.3

## Data Availability

The experimental dataset for this study is from the China Power Line Insulator Dataset (CPLID), and collected images of insulator defects in actual production, as well as from the NEU-DET dataset.
